# Polystyrene Nanoplastic Exposure Causes Reprogramming of Anti-Oxidative Genes *Hmox1* and *Sod3* by Inhibiting Nuclear Receptor RORγ in the Mouse Liver

**DOI:** 10.3390/biology15020135

**Published:** 2026-01-13

**Authors:** Pingyun Ding, Madesh Muniyappan, Chuyang Zhu, Chenhui Li, Saber Y. Adam, Yu Wang, Thobela Louis Tyasi, Peng Yuan, Ping Hu, Haoyu Liu, Demin Cai

**Affiliations:** 1 School of Pharmacy & School of Biological and Food Engineering, Changzhou University, Changzhou 213000, China; dpy@cczu.edu.cn (P.D.); wangyu@cczu.edu.cn (Y.W.); 2 Jiangsu Key Laboratory of Animal Genetic Breeding and Molecular Design, College of Animal Science and Technology, Yangzhou University, Yangzhou 225009, Chinazhuchuyang@foxmail.com (C.Z.);; 3 Department of Agricultural Economics and Animal Production, School of Agricultural and Environmental Sciences, University of Limpopo, Private Bag X1106, Polokwane 0727, South Africa

**Keywords:** polystyrene nanoplastics, *Hmox1*, oxidative stress, RORγ, histone modifications

## Abstract

Plastic pollution is acknowledged as a serious problem for ecosystems. Among these plastics, polystyrene nanoplastics (PS-NPs) are emerging environmental pollutants, and their biological effects on hepatotoxicity are the least explored. Therefore, the present work examined the effect of PS-NPs on the hepatic transcription of the antioxidant genes *Hmox1* and *Sod3* in mice (*n* = 6, treatment (PS-NPs) vs. vehicle group (Veh)), mediated by RORγ and epigenetic modifications. The results show that PS-NP mice had significantly reduced body weight; increased activity of adenosine triphosphate (ATP), superoxide dismutase (SOD), catalase (CAT), glutathione peroxidase (GSH), and Complexes I, III, and V in the liver; and increased Alanine Transaminase (ALT), Aspartate Transaminase (ASP), Alkaline Phosphatase (ALP), malondialdehyde (MDA) and reactive oxygen species (ROS) compared to the Veh group. Furthermore, PS-NPs resulted in considerably lower relative mRNA expression of Hmox1, Sod3, and RORγ in the liver than the Veh group. Likewise, when compared to Veh, PS-NPs significantly reduced the enrichment of RORγ, as well as the occupancies of the key components of the transcriptional activation pathway (P300, SRC1, Pol II, Ser5-Pol II, and Ser2-Pol II) at the loci of Hmox1 and Sod3. In comparison to Veh, PS-NPs showed downregulated occupancies of the histone active marks H3K9ac and H3K18ac, while H3K4me3 and H3K27me3 were higher at the target loci of Hmox1 and Sod3. In conclusion, the present study highlights that PS-NPs induce oxidative stress by modifying Hmox1 and Sod3 in mice’s livers through histone changes and nuclear receptor RORγ modulation.

## Simple Summary

Polystyrene nanoplastics (NPs) are emerging environmental contaminants that may pose serious health hazards to human health. There is a remarkable lack of comparable research in mammalian models, although systemic toxicity studies have been carried out on a variety of aquatic species and poultry. This study investigated organ damage caused by NPs in mice through a comprehensive examination of biological parameters. The current work also demonstrates that PS-NPs cause oxidative stress in mice’s livers by altering levels of Hmox1 and Sod3 through histone modifications and nuclear receptor RORγ regulation. The findings of the study provide a comprehensive theoretical basis for the toxicity of PS-NPs and a potential therapy avenue for PS-NP-induced health damage.

## 1. Introduction

Polystyrene (PS), produced via the polymerization of styrene monomers, is one of the most widely used plastics due to its inert nature and versatility [1]. It is commonly found in toys, household appliances, food packaging, and electronics [2]. The breakdown and replacement of these products continuously generate plastic waste, with global emissions projected to reach 53 million tons annually by 2030 [3]. Owing to its extensive use, PS is frequently identified as a major component of plastic waste in airborne sediments and marine environments. Such waste can fragment into microplastics (MPs, diameter of less than 5 mm) and nanoplastics (NPs, diameter < 100 nm), which are also inadvertently generated during production and processing [4]. These particles enter the human body through skin contact, inhalation, and ingestion [5], and have been detected in diverse biological materials, including blood, placenta, breastmilk, feces, and various tissues [6], raising significant public health concerns.

NPs and MPs exert harmful effects across animal species, including growth retardation, metabolic disruption, intestinal damage, and immune, reproductive, and neurotoxicity [7,8]. Long-term exposure to these particles, which can accumulate chemical pollutants such as polychlorinated biphenyls and polycyclic aromatic hydrocarbons, has been linked to neurological diseases, cancer, and hemolytic anemia in humans [9,10]. Their small size and persistence enhance their ability to cross biological barriers, accumulate in organs, and cause systemic harm [11]. The liver, a central organ for metabolism and immunity, is a key target [12,13]. Oral or inhalation exposure to PS-NPs in mice induces hepatocyte apoptosis, lipid metabolic disturbances, fibrosis, and inflammation [14]. Likewise, PS-NPs have the potential to be hepatotoxic via inhalation routes [14]. However, the specific molecular mechanisms underlying PS-NP-induced hepatotoxicity remain unclear.

Recent studies highlight the important role of the nuclear receptor retinoic acid receptor-related orphan receptor-gamma (RORγ), encoded by the *RORC/NR1F3* gene, in regulating immunity, metabolism, circadian rhythm, and disease progression [15,16]. Though initially characterized as an orphan receptor, RORγ may be activated by cholesterol synthesis intermediates and bile acid metabolites [17]. It is expressed in multiple tissues, including the liver, and helps regulate circadian gene expression and antioxidant responses involving glutathione (GSH), superoxide dismutase (SOD), and catalase (CAT) [18,19,20].

Gene expression is also modulated by epigenetic mechanisms such as histone methylation, acetylation, and phosphorylation, which alter chromatin structure and accessibility [21]. In particular, histone acetylation in lysine residues on histones H3 and H4 is associated with transcriptionally active chromatin and can be influenced by nuclear receptors like RORγ [22]. Exposure to PS-NPs has been shown to alter DNA methylation patterns in liver cells, affecting genes involved in inflammation, detoxification, and lipid metabolism, processes linked to liver fibrosis and non-alcoholic fatty liver disease (NAFLD) [23,24]. PS-NPs may also modify histone landscapes, thereby influencing chromatin structure and the binding of transcriptional machinery such as RNA polymerase II (Pol II) [25]. Dynamic histone modifications are particularly crucial for regulating rhythmic gene expression. Nevertheless, the impact of PS-NPs on the epigenetic regulation of core antioxidant genes remains unexplored.

In the current study, we investigated the effects of PS-NPs on the programming of two critical hepatic antioxidant genes, *Hmox1* and *Sod3.* We aimed to examine whether PS-NPs alter their expression through mechanisms involving RORγ and associated epigenetic modifications.

## 2. Materials and Methods

### 2.1. PS-NP Sources

The Baseline ChromTech Research Center (Tianjin, China) provided PS-NPs (80 nm, 10 mg/mL), which were then used in animal toxicological tests. The standard diet was purchased from Jiangsu Xietong Pharmaceutical Bio-Engineering Co., Ltd. (Jiangning, Nanjing, Jiangsu, China) (Table 1).

### 2.2. Animals

Six-week-old male C57BL/6 mice were purchased from Yangzhou University’s Model Animal Center in Yangzhou, China. Following a 7-day acclimatization period, twelve mice were randomly assigned to two groups (*n* = 6 per group): a vehicle control group, which received phosphate-buffered saline (PBS), and a PS-NP-exposed group, which received 80 nm-sized PS-NPs. For 28 days, mice in the PS-NP group were administered 200 µL of a PS-NP suspension (10 mg/mL) via oral gavage once daily, delivering a dose of 1 mg per mouse per day, as adapted from previous studies [26,27,28]. Mice in the control group received an equivalent volume (200 µL) of PBS via the same route. This exposure schedule was created to replicate, in a controlled laboratory environment, a realistic situation of ingesting nanoplastics. Yangzhou University’s Animal Welfare and Ethics Committee (SYXK [Su] 2023-0089) approved the animal studies on 26 February 2024, and they were carried out in accordance with the guidelines of the Yangzhou University Institutional Animal Care and Use Committee (IACUC). At the end of the experiment, blood samples were collected for serum extraction, and liver tissues were collected.

### 2.3. Complete Blood Count (CBC) and Liver Function Test

The 16 standard complete blood count (CBC) parameters (red blood cells, white blood cells, platelets) were measured using a Sysmex XN-9000 automated hematological analyzer (Sysmex Corporation, Tokyo, Japan) according to the manufacturer’s instructions. The International Council for Standardisation’s guidelines were used to evaluate the precision and accuracy of the hematology analyzer [29]. Before and following measurement, high and low levels of quality control were carried out.

Blood was centrifuged for 10 min, yielding 12,000 *g* of serum, which was then kept at −20 °C until analysis. By measuring the activity of essential liver enzymes, hepatic function was assessed. Using assay kits (Nanjing Jiancheng Bioengineering Company, Nanjing, China), serum levels of Alanine Transaminase (ALT, Ab105134), Aspartate Transaminase (AST, Ab105135), and Alkaline Phosphatase (ALP, ARG81296) were determined following the manufacturer’s instructions.

### 2.4. Hematoxylin and Eosin Staining

The liver tissues were fixed in 4% paraformaldehyde solution for 48 h and then embedded in paraffin and sliced into 5 μm sections that were stained with hematoxylin and eosin. The histopathological changes in liver tissues were observed using a microscope (DXM12000F; Nikon, Tokyo, Japan).

### 2.5. DNA Content

The detection of DNA damage was conducted using the PCR-based methodology established by Gu et al. [21]. DNA was extracted utilizing a DNeasy Tissue Kit (Beyotime, Shanghai, China), following the manufacturer’s guidelines. The quality and amount of DNA were assessed by Nanodrop 260 nm analysis (Thermo Fisher Scientific, Waltham, MA, USA). DNA was standardized to 10 ng µL^−1^ for all samples, and 1 µL was utilized in real-time PCR experiments. The PCR reactions, with a final volume of 15 µL, comprised 7.5 µL of RealQ Plus 2 × Master Mix Green (Ampliqon Biotechnology company, Stenhuggervej 22, 5230 Odense, Denmark), 0.2 µM of primer (refer to Table 1), and 10 ng of normalized DNA, and they were conducted in triplicate. Thermal cycling was conducted for 15 min at 94 °C, succeeded by 40 cycles of 15 s at 94 °C, 30 s at 60 °C, and 30 s at 72 °C. Melt curve analysis was conducted on reactions at temperatures between 70 and 95 °C. The Ct value from real-time PCR reactions was utilized for quantifying DNA damage, employing the 2^−ΔΔct^ calculation to determine alterations in DNA copy number relative to the control samples.

### 2.6. Caspase 3/7 Activity

The caspase 3/7 activity assay uses caspase 3/7 to catalyze the substrate Ac-DEVD-pNA, producing yellow pNA (p-Nitroaniline). The pNA exhibits absorption around 405 nm; the activity of caspase 3/7 was quantified by measuring the absorbance at this wavelength. Both groups underwent evaluation with a caspase 3/7 activity assay kit.

### 2.7. Oxidative and Antioxidant Indexes

Initially, the tissues were homogenized and then resuspended in PBS, after which the supernatant was collected. A Total Superoxide Dismutase Assay Kit with WST-8 (Beyotime, S0101S, Shanghai, China) and a Catalase Assay Kit (Beyotime, S0051, Shanghai, China) were utilized to assess the enzyme activity of SOD and CAT. The absorbances of SOD and CAT were recorded at 450 nm and 520 nm, respectively. The contents were determined based on the standard curve and subsequently standardized to protein concentrations. Assay kits for GSH and glutathione disulfide (GSSG) (Beyotime, S0053, Shanghai, China) were utilized to quantify the GSH content, which was subsequently standardized to tissue weight. Using the specified treatments, the Lipid peroxidation (LPO) Malondialdehyde (MDA) Assay Kit (Beyotime, S0131S, Shanghai, China) was employed to measure the MDA levels. Briefly, the samples underwent centrifugation following treatment with lysis buffer. The supernatants were subsequently combined with TBA detection solution to assess the absorbance at 532 nm, allowing for the calculation of MDA contents based on the standard curve. Ultimately, the data were normalized to tissue weight. Concentrations were assessed utilizing commercial kits sourced from Nanjing Jiancheng Bioengineering Institute (Nanjing, China).

### 2.8. ROS Levels Assay

The analysis utilized an OxySelect In Vitro ROS/RNS Assay Kit (Cell Biolabs, STA-347, San Diego, CA, USA). Highly sensitive dichloro-dihydro-fluorescein (DCFH) was primed for a non-fluorescence assay of DCFH-DiOxyQ. Intensely fluorescent DCF, generated by the oxidation of DCFH by reactive oxygen species (ROS), was detected at an excitation wavelength of 480 nm and an emission wavelength of 530 nm to quantify ROS levels in liver tissues.

### 2.9. Activity of Hepatic Complexes I, III, and V and ATP Content Assay

Tissues were initially homogenized and subsequently washed in PBS. The supernatant was then obtained, and the activity of mitochondrial respiratory chain Complexes I, III, and V was assessed using suitable commercial assay kits (Comin Technologies, Co., Ltd., Suzhou, China). Liver adenosine triphosphate (ATP) concentration was quantified with an ATP assay kit (Beotime, S0026, Shanghai, China). The absorbance of ATP was recorded at 450 nm.

### 2.10. Western Blotting Analysis

Mouse liver tissues were homogenized in RIPA lysis buffer by adding Phenylmethylsulfonyl fluoride (PMSF) and phosphatase inhibitors and using bicinchoninic acid (BCA) protein assay kits (Pierce, Rockford, IL, USA). A total of 40 μg of protein was resolved using 10% SDS-PAGE and subsequently transferred to PVDF membranes, which were blocked with tris-buffered saline Tween 20 (TBST) containing 5% non-fat milk and kept at room temperature for 2 h, whereas phosphorylated proteins were blocked with 1% bovine serum albumin (BSA). The membranes were then treated with the appropriate primary antibodies (GAPDH, 1:10,000; RORγ, 1:4000; Sod3, 1:5000; Hmox1, 1:4000) at 4 °C overnight. Subsequent to washing with TBST, the membranes were incubated with HRP-conjugated secondary antibody (1:10,000) at ambient temperature, followed by further washing. The bands were visualized using an ECL chemiluminescence kit, and the grayscale values of the bands were analyzed using IPP version 8 software.

### 2.11. Total RNA Isolation and Real-Time PCR

Total RNA was extracted utilizing Trizol (Invitrogen, Waltham, MA, USA) in accordance with the manufacturer’s guidelines and stored at −80 °C. Thereafter, the RNA was reverse-transcribed into cDNA following the provided instructions (Vazyme, Nanjing, China). mRNA expression was assessed using the guidelines provided by Vazyme, Nanjing, China, and its relative expression was computed utilizing the 2^−ΔΔCT^ method.

### 2.12. Gene Enrichment Analysis (GSEA) of RNA-Seq

The enriched pathway profiles were determined utilizing the Gene Set Enrichment Analysis (GSEA) 4.1.0 software. Furthermore, statistically significant biological processes or pathways in differentially expressed genes (DEGs) within the GO and KEGG pathways were evaluated and categorized utilizing the Metascape database (http://metascape.org/gp) and DAVID (https://davidbioinformatics.nih.gov/) accessed on 23 May 2025. GSEA enrichment analysis plots, KEGG enrichment bubble plots, cnet plots, volcano plots, and GO-pathway enrichment result circle plots were generated utilizing an online data analysis and visualization platform (http://bioinformatics.com.cn, accessed on 23 May 2025).

### 2.13. ChIP-qPCR Measurement

Small pieces of liver tissue were sliced, fixed for five minutes in 1% formaldehyde, and subsequently fixed for five minutes in ice-cold glycine. Subsequently, the samples were resuspended in a lysis buffer composed of 50 mM HEPES with the following specifications: pH 8.0, 140 mM NaCl, 1 mM EDTA, 10% glycerol, 0.5% NP-40, and 0.25% Triton X-100. Following cleaning, the samples were suspended in cutting buffer (pH 8.0, 0.1% SDS, 1 mM EDTA, and 10 mM Tris-HCl) and subjected to sonication with a Covaris E220, (Covaris, LLC., Woburn, MA, USA) in accordance with the manufacturer’s instructions. Protein-G magnetic beads were used to treat crude chromatin fragments after they had been exposed to specific antibodies for an entire night at 4 °C. Purified ChIP-DNA is required for ChIP-qPCR, the next stage of library creation. With the following adjustments, chromatin immunoprecipitation was performed as previously described [21]. Coarse chromatin extracts from liver tissue were removed by binding magnetic beads (Thermofisher Scientific, 10004D, Waltham, MA, USA) after they had been treated with immune serum for two hours at 4 °C. After that, the pretreatment chromatin solutions were incubated with the designated antibodies for one more night at 4 °C. After blocking the protein A beads with BSA, the samples were precipitated by adding sonicated salmon sperm DNA. Before ChIP preparations, the 20 mM dithiothreitol solution was eluted, vortexed, and diluted for 0.5 h at 37 °C to produce immunoprecipitated complexes. Following an overnight incubation period at 4 °C with the designated antibodies for secondary ChIP, qRT-PCR was used to evaluate the ChIP-ed DNA.

### 2.14. Statistical Analysis

All data analysis was conducted using GraphPad Prism (version 9.0), following normality assessments and evaluations of variance homogeneity. The results were given as means ± standard deviation (SD). Statistical analysis was conducted using two-tailed non-parametric *t* tests. Significance was defined as *p* < 0.05

## 3. Results

### 3.1. PS-NP Supplementation Causes Liver Injury in Mice

**Figure 1:**
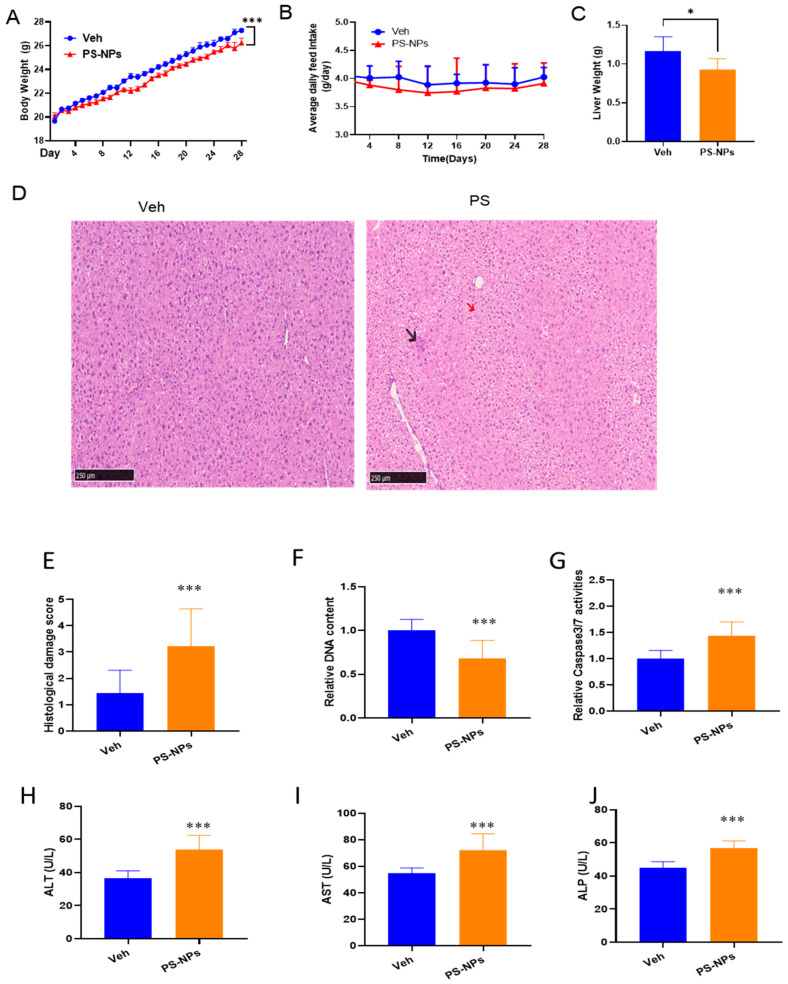
The effects of PS-NP oral administration on body and liver weight, histological changes, and liver enzyme biomarkers in mice. (**A**) Body weight (g). (**B**) Average daily feed intake (g). (**C**) Liver weight (g). (**D**) Liver histological changes in H&E staining. (**E**) Histological damage score. (**F**) Relative caspase3/7 activity. (**G**) Relative DNA content. (**H**) Alanine Transaminase (ALT) levels. (**I**) Aspartate aminotransferase (AST) levels. (**J**) Alkaline Phosphatase (ALP) levels. The data is presented as means ± SD (*n* = 6). * *p* < 0.05, *** *p* < 0.001.

Essential phenotypic differences between the Veh and the PS-NP groups were determined, where mice from the PS-NP group exhibited a decrease in body weight of 10% and a reduction in average daily feed intake of 8% compared to the Veh group (Figure 1A,B). Compared with the Veh group, the liver weight in the PS-NP group was decreased by 19% (Figure 1C). The impacts of PS-NPs on the target organs were further investigated. Histological analysis of murine liver tissues subjected to the PS-NP treatment showed immune cell infiltration, hepatocyte vacuolization and displaced nuclei, hepatocyte shrinkage with pyknotic nuclei, and an increase in sinusoidal spaces (Figure 1D). Consistent with these observations of liver tissue pathology, PS-NPs significantly increased the average histological damage score by 22.7% compared with the Veh group (Figure 1E). Accordingly, assessment of apoptosis revealed a 38% increase in caspase 3/7 activity and a 29.4% decrease in DNA content in the livers of PS-NP-exposed mice compared with the Veh group (Figure 1F,G). Next, serum biochemical indicators and the hematological system were investigated (Table 2). The study showed that WBCs and monocytes increased by 17.77% and 30%, respectively, and platelet count decreased by 18.48% in mice exposed to PS-NPs. In contrast, no significant changes were observed in other serum biochemical indices. Moreover, liver ALT, AST, and ALP levels were increased by 39.4%, 31.6%, and 25% in the mice exposed to PS-NPs compared with the healthy control (Figure 1H–J).

### 3.2. PS-NPs Induce Oxidative Stress in Murine Livers

**Figure 2:**
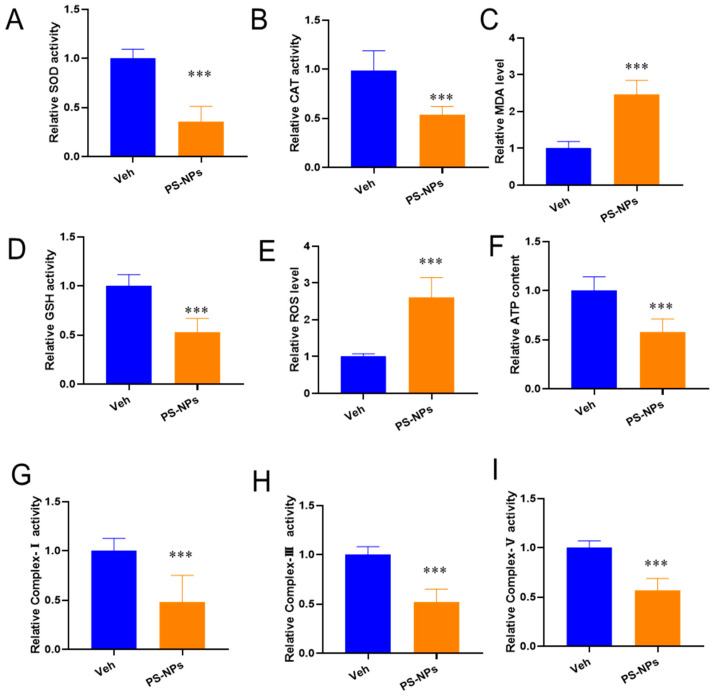
Oxidative stress was observed after being challenged with PS-NPs. The relative activity of (**A**) superoxide dismutase (SOD), (**B**) catalase (CAT), (**C**) malondialdehyde (MDA), and (**D**) glutathione peroxidase (GSH). The relative levels of (**E**) reactive oxygen species (ROS) and (**F**) adenosine triphosphate (ATP) content. The relative activity of (**G**) mitochondria enzyme Complex I, (**H**) Complex III, and (**I**) Complex V. The data are presented as means ± SD (*n* = 6). *** *p* < 0.05.

To verify whether PS-NPs induced oxidative stress in the liver, the associated enzymes were examined. The results showed that the antioxidative enzymes SOD, CAT, and GSH were decreased by 21.42%, 29.12%, and 23.02%, and MDA was increased by 35.15%, respectively, in mice challenged with PS-NPs compared with the Veh group (Figure 2A–D). In contrast, the results showed that PS-NPs led to 45.87% upregulation of ROS production in mice challenged with PS-NPs (Figure 2E). Furthermore, it was shown that exposure to PS-NPs decreased the level of ATP levels and the enzyme activity of mitochondrial Complexes I, III, and V by 23.45%, 31.23%, 29.67%, and 31.41%, respectively relative to the control (Figure 2F–I). All of these findings suggest that the PS-NP exposure resulted in oxidative stress in the murine liver.

### 3.3. Anti-Oxidative Genes Are Susceptible to PS-NP Exposure

**Figure 3:**
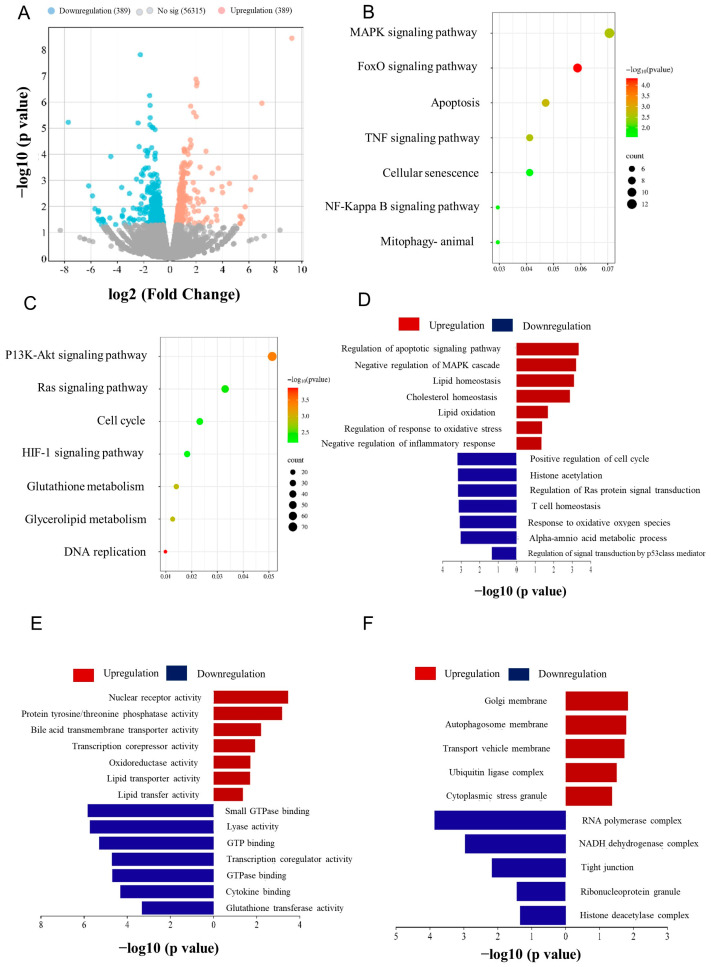
The transcriptional profiling showed that the anti–oxidative pathway was modulated by challenge with PS-NPs. (**A**) Volcano plot visualization of the differential gene expression profiles between the PS-NPs and the vehicle group by transcriptome analysis. (**B**,**C**) DEGs involved in the inflammation and apoptosis pathways were the most abundant downregulated enriched DEGs analyzed by KEGG. (**D**–**F**) Genes involved in the inflammation and apoptosis pathways were among the most enriched among the pathways analyzed by GO.

The effects of PS-NP exposure on the primary transcription programs of the liver were examined using RNA-seq analysis. Hepatic transcriptome analysis showed that PS-NP challenge alters the essential transcriptional pathways. In total, 689 differentially expressed genes (DEGs, |Log2 (fold change) | > 1, *p* < 0.05) were identified between the PS-NP and Veh groups, comprising 300 downregulated and 389 upregulated genes (Figure 3A). GO and KEGG pathway enrichment showed significant enrichment in genes of the inflammation and apoptosis pathways. GSEA revealed that DEGs significantly downregulated hallmarks associated with the cell cycle, apoptosis, oxidative stress, lipid oxidation, and inflammatory response pathways (Figure S1A–E).

**Figure 4:**
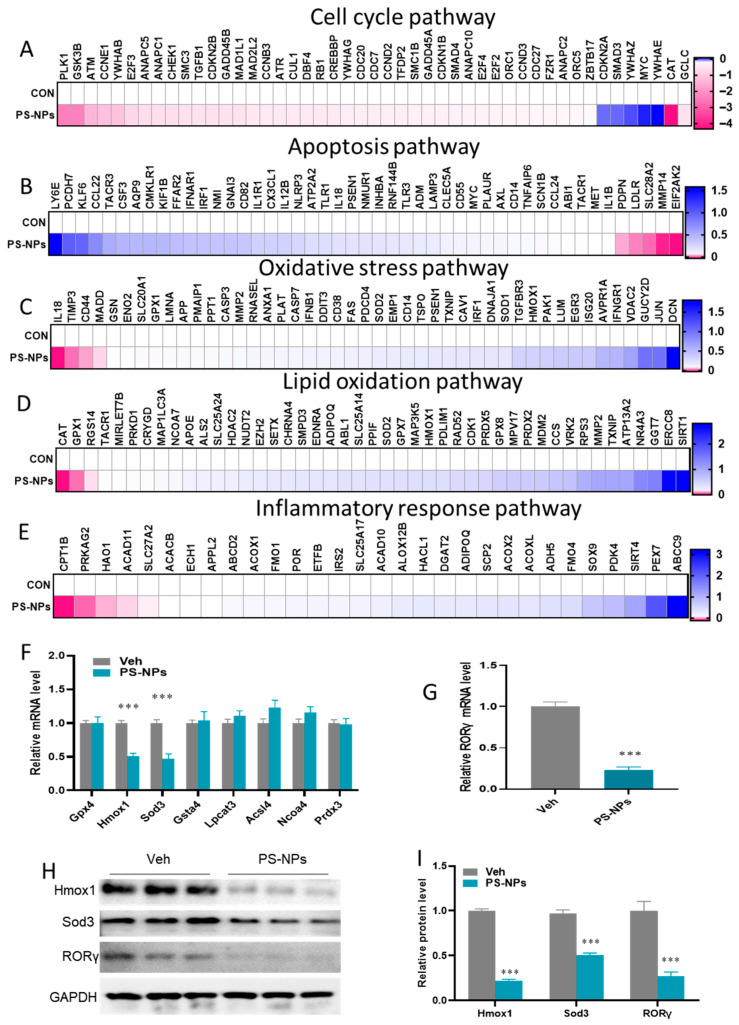
The expression of RORγ in the livers was dysregulated after they were challenged with PS-NPs. Heatmap showing the relative mRNA levels of key genes in the (**A**) cell cycle pathway, (**B**) apoptosis pathway, (**C**) oxidative stress pathway, (**D**) lipid oxidation pathway, and (**E**) inflammatory response pathway. (**F**) mRNA expression changes in the antioxidant-related genes. (**G**) Variations in RORγ mRNA expression. (**H**,**I**) Western blotting analysis was performed to evaluate the expression of nuclear RORγ, *Hmox1*, and *Sod3* at the protein level. The data is presented as means ± SD (*n* = 6). *** *p* < 0.001.

The pathway-focused genes subset showed that most cell cycle, apoptosis, oxidative stress, lipid oxidation, and inflammatory response pathways were significantly altered in GO and KEGG pathway enrichment due to PS-NP exposure (Figure 4A–E). Next, the study focused on the anti-oxidation enzymes against oxidative stress using a pathway-focused data analysis. The qRT-PCR analysis confirmed that the key antioxidant genes, including *Hmox1* and *Sod3*, were downregulated by 45.34% and by 48.62%, respectively, in the livers of mice challenged with PS-NPs compared with the Veh group (Figure 4F). We found a significant difference between RORγ gene expression as evaluated by qRT-PCR and RNA-seq analysis, showing a 19.34% reduction in mRNA expression levels (Figure 4G). Consistently, the decrease in Hmox1, Sod3, and RORγ expression at the protein level was confirmed by immunoblotting (Figure 4H,I and Figure S2).

### 3.4. Loss of RORγ Binding to the Enhancers and the Promoters of the Hmox1 and Sod3 Genes

**Figure 5:**
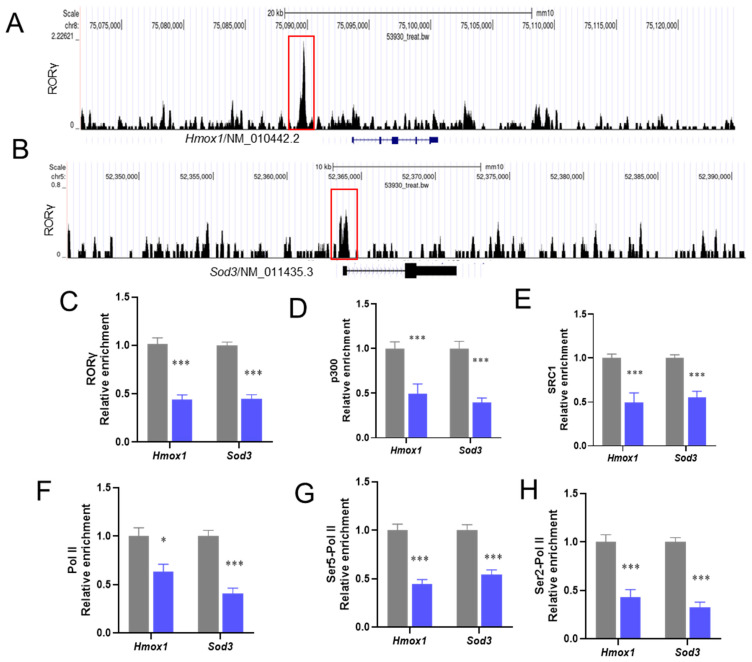
RORγ binding occupancies were reduced in mice challenged with PS-NPs. (**A**,**B**) ChIP–seq signal visualization of RORγ at the loci of representative antioxidant metabolism genes *Hmox1* and *Sod3*; the numbers coupled with the group names of Veh or PS-NPs represent the maximum range of data. (**C**–**H**) ChIP-qPCR analyses of RORγ, P300, SRC1, Pol II, Ser5-Pol II, and Ser2-Pol II occupancies at the loci of *Hmox1* and *Sod3*. The data is presented as means ± SD (*n* = 6). * *p* < 0.05, *** *p* < 0.001.

**Figure 6:**
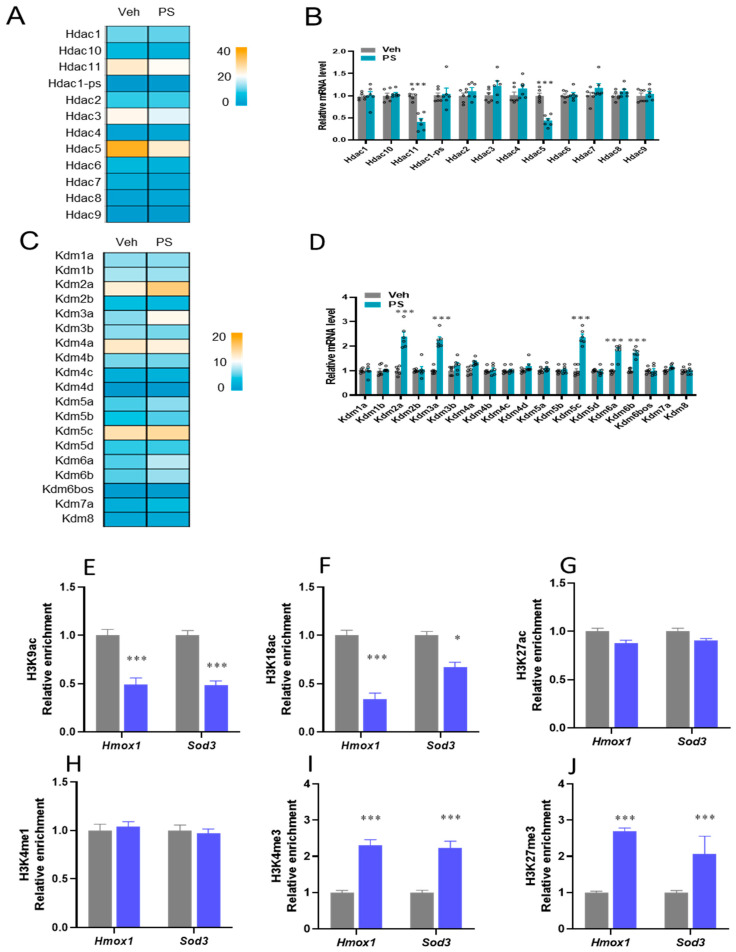
Ps-NP exposure modifies histone modification at the loci of *Hmox1* and *Sod3*. (**A**) The heat map of *HDAC* genes. (**B**) The relative mRNA levels of HDAC genes. (**C**) The heat map of KDM genes. (**D**) The relative mRNA levels of KDM genes. (**E**–**J**) The relative enrichment of the occupancy of histone marks (H3Kac, H3K18ac, H3K27ac, H3K4me1, H3K4me3, and H3K27me3) was analyzed by ChIP-qPCR. The data is presented as means ± SD (*n* = 6). * *p* < 0.05, *** *p* < 0.001.

Subsequently, the study examined the genes in murine livers that were most affected by exposure to PS-NPs. ChIP-seq analysis showed RORγ binding to the enhancer’s key antioxidant genes, including *Hmox1* and *Sod3* (Figure 5A,B). Further ChIP-qPCR analysis confirmed these results, showing enrichment of *RORγ*, *P300*, *SRC1*, Pol II, Ser5-Pol II, and Ser2-Pol II at the target loci of antioxidant genes (Figure 5C–H). The enrichment of RORγ at the target loci of *Hmox1* and *Sod3* was significantly reduced in mice challenged with PS-NPs compared with the Veh group. In agreement with the reduced binding of RORγ, the occupancies of co-activators P300 and SRC1 were also decreased in the PS-NP-treated mice. The transcriptional inhibition was validated by the lower levels of binding of Pol II, SER5-Pol II, and SER2-Pol II at both initiation and elongation (Figure 6E–J).

### 3.5. Histone Modifications Facilitate RORγ-Driven Hmox1 and Sod3 Reprogramming

The study further showed altered profiles of the genes encoding histone deacetylases (HDACs) and histone lysine demethylases (KDMs), including Hdac11, Hdac3, Hdac5, Kdm2a, Kdm3a, Kdm5c, Kdm6a, and Kdm6b, as determined by transcriptomic analysis. Decreased Hdac11 and Hdac5, along with increased Kdm2a, Kdm3a, Kdm5c, Kdm6a, and Kdm6b, in the PS-NP mice were validated by qRT-PCR (Figure 6A–D). These typical expressions of the genes involved in HDACs and KDMs suggest that epigenetic regulators are involved in the modulation of nuclear receptor RORγ. ChIP-qPCR was used to investigate the histone mark enrichments at the loci of *Hmox1* and *Sod3* genes since epigenetic regulators are involved in the modulation of nuclear receptors. The histone marks H3K9ac and H3K18ac, related to transcriptional activation, were significantly downregulated, whereas H3K4me3 and H3K27me3, related to transcriptional activation, were significantly upregulated by PS-NP exposure in mice; however, this was not the case for H3K27ac and H3K4me1, as enhancers of *Hmox1* and *Sod3*, respectively (Figure 6E–J).

## 4. Discussion

As one of the major components of plastic, PS-NPs have become a serious environmental and health hazard because of their microscopic size and bio-accumulative characteristics [30]. This study examined the toxicological impacts of PS-NPs ingested via drinking water on the programming of antioxidant *Hmox1* and *Sod3* genes in the mouse liver through RORγ and epigenetic changes in mice. It was found that mice exposed to PS-NPs showed suppression in body weight, which was also seen in earlier studies on mice exposed to PS-NPs at doses ranging from 0.01 mg/mL to 1 mg/mL [31]. Indeed, PS-NPs can penetrate the intestinal barrier, build up in organs such as the brain, liver, and kidneys through the oral route, and cause metabolic alterations that vary depending on the concentration and type of polymer [32]. In the current study, it was determined that the liver was the main target of PS-NPs, as shown by a decreased liver weight and body weight. Histological examinations and liver function indicators supported this by showing increased caspase3/7, loss of DNA content, and elevated ALT, AST, and ALP—liver impairment biomarkers. Accordingly, reduced liver function and damage were also shown in response to PS-NP exposure, manifesting as elevated serum levels of ALT and AST [33,34]. On the other hand, it was suggested that PS-NPs can postpone liver development instead of immediately causing liver toxicity. This aligns with research showing microplastics can cause oxidative damage to fish livers without histological damage [35]. The discrepancy may therefore result from variations in PS-NP dosages and administration routes. PS-NPs mainly raise the levels of liver ALT, AST, and ALP in mice by causing oxidative stress, inflammation, and cellular disruption, which damages the hepatocytes and causes these enzymes to be released into the bloodstream [33,36,37]. In this study, mice subjected to chronic exposure to PS-NPs consistently showed significantly increased levels of ALT, AST, and ALP, accompanied by histopathological characteristics including ballooning degeneration of hepatocytes, nuclear pyknosis, and infiltration of inflammatory cells.

In mice, PS-NPs can alter the CBC profile, impacting several hematological parameters [38]. In the present study, PS-NP exposure in mice led to a significant rise in WBCs and monocytes, but a decrease in PLT count, when compared to healthy control mice. These results were consistent with a study that found that exposure to PS-NPs reduced WBC counts, suggesting possible immunosuppression [39]. PS-NPs may also cause an inflammatory reaction that raises specific WBC types [14]. Meanwhile, a study using single-cell mass cytometry revealed that NPs accumulate in different immune cell subpopulations, reducing cell survival and functionality [40]. Nevertheless, the effects vary according to the concentration and duration of PS-NP exposure, and the mechanisms that mediate these effects are associated with oxidative damage, inflammation, direct contact with blood cells, and different cell death pathways [30,41,42].

PS-NPs can cause oxidative stress via intricate regulation, by increasing the formation of reactive oxygen species (ROS), disrupting calcium homeostasis, and inhibiting antioxidant enzymes, as well as causing mitochondrial malfunction in a variety of cell types [30,43]. It is demonstrated that oxidative stress is strongly associated with increased hepatic CYP2E1 levels, which affects mitochondrial function, glycolipid metabolism, and hepatic insulin signaling. Whereas 80 nm NPs lower NR PPARα activation, which in turn reduces mitochondrial fatty acid β oxidation and increases liver steatosis [44]. Accordingly, this study revealed that PS-NPs upregulated the expression of the oxidative stress genes *MDA* and *ROS* while downregulated the expression of ATP, SOD, CAT, and GSH in the murine liver, which supports earlier research showing that exposure to PS-NPs increased oxidative stress in the liver [45]. In addition, exposure to 80 nm PS-NPs markedly reduced the activity of antioxidant enzymes (CAT, SOD, and GSH Px) and increased MDA levels in human gastric epithelial cells [46,47]. In contrast, it is reported that livers exposed to PS-NPs at a dose of 0.5 µm did not exhibit significant oxidative stress, as indicated by unchanged GPX, MDA, and SOD protein levels, which, again, may result from variations in the PS-NP dosages and particle sizes.

RNA-Seq analysis was used to gain deeper insight into the molecular events occurring in the liver of mice exposed to PS-NPs. Our results revealed relevant pathways linked to oxidative stress, lipid oxidation, inflammatory response, cell cycle, and apoptosis. Among these, heme oxygenase 1 (encoded by *Hmox1*) is a vital antioxidant enzyme that breaks down heme into carbon monoxide, free iron, and biliverdin to control oxidative stress [48], and has been implicated in several inflammatory diseases, such as metabolic syndrome, neurological conditions, and cardiovascular diseases, to restore redox equilibrium [49,50,51]. It has been demonstrated that Hmox1 activates the Nrf2 signaling pathway, which increases the expression of several cytoprotective and antioxidant genes. When Nrf2 is activated, GSH and SOD may be produced, thereby counteracting the generation of ROS [48]. Hmox1 induction can lessen oxidative damage caused by a variety of toxicants, including those induced by exposure to PS-NPs. It is reported that PS-NPs can cause oxidative stress in the liver, which activates the NRF2-NLRP3 signaling pathway and influences the expression of Hmox1 [33]. Meanwhile, extracellular superoxide dismutase, or Sod3, is essential for scavenging superoxide radicals and converting them into oxygen and hydrogen peroxide [52]. When PS-NP stress the liver, Sod3 expression and activity are also involved in regulating oxidative balance [53]. Our observations indicated that the PS-NPs inhibited the transcription of Hmox1, suggesting that Sod3 may emerge as a key factor associated with PS-NPs.

It has been demonstrated that RORγ can directly interact with the genes of the MVA pathway, facilitating the transcription of relevant genes [54]. In our study, it is demonstrated that PS-NP exposure significantly diminished RORγ enrichment at the loci of the critical genes *Hmox1* and *Sod3*, which was evidenced by ChIP-Seq analysis, confirming that RORγ directly interacts with the core genes associated with the anti-oxidation process, suggesting that the reduced binding enrichment of RORγ as a transcriptional regulator led to the downregulation of *Hmox1* and *Sod3* gene expression. Through ChIP-re-ChIP analysis, we further demonstrate that RORγ binding is concentrated at the same locations of the *Hmox1* and *Sod3* genes, whereas PS-NPs decreased the occupancies of *Hmox1* and *Sod3* on these targets utilizing the RORγ-ChIP-ed DNA. This provides compelling evidence that Nrf2 is involved in RORγ’s actions through the reciprocal cross-talk between these two modulators. Pharmacological activation of RORγ has been proposed to reduce PS-NP-associated inflammatory responses [21]. We noted a PS-NP-induced redox signaling alteration, which may have been controlled by RORγ and its physical interaction with related factors.

Additionally, since RORγ controls both lipid metabolism and inflammatory responses [53]. Its downregulation by PS-NPs could contribute to the development of liver metabolic disorders. Indeed, it was discovered that PS-NP-exposed mice had lower levels of *Hmox1*, *Sod3*, and *RORγ* expression than control animals. Direct binding of RORγ to MVK pathway genes results in transcription [55]. Meanwhile, P300 has been shown to act as a transcription coactivator, facilitate chromatin decondensation, acetylate core histones, etc. [56]. Acetylation is thought to play a role in the Nrf2-dependent oxidative response, as it was found that P300 acetylates Nrf2 and improves promoter-specific DNA binding during oxidation [57]. To facilitate binding, steroid receptor coactivator 1 (SRC-1) adds basic amino acid residues to each DNA helix. Furthermore, a region seen in most SRCs interacts with p300/CBP by binding CBP, and is commonly found in the p300/CBP epigenetic regulatory complex [58]; together, the above contribute to the formation of the basal transcription machinery. This study revealed that PS-NP mice had lower protein levels of RORγ, P300, and SRC-1 at the target loci of *Hmox1* and *Sod3*. Meanwhile, phosphorylation of Pol II’s C-terminal domain (CTD), which contains repetitive heptapeptide sequences (Tyr1-Ser2-Pro3-Thr4-Ser5-Pro6-Ser7), controls transcription [59,60]. In particular, elongation is related to Ser2 phosphorylation, whereas transcription initiation is linked to Ser5 phosphorylation [21]. At various phases, transcription-associated proteins are recruited by these phosphorylation processes [61]. PS-NP mice in our study showed a decrease in Pol II, as well as Ser5-Pol II and Ser2-Pol II elongation, at the target loci of *Hmox1* and *Sod3*. Disrupted recruitment or activity of kinases responsible for these phosphorylation processes may be the cause of the decrease in Pol II and decreased Ser5 and Ser2 phosphorylation at the *Hmox1* and *Sod3* locus. For example, Pol II elongation will be impacted if exposure to PS-NPs reduces the recruitment or activity of CDK7, which is in charge of Ser5 phosphorylation, or CDK9/CDK12, which is in charge of Ser2 phosphorylation [62,63]. Decreased transcription of *Hmox1* and *Sod3* results from reduced Pol II activity and changed phosphorylation patterns, which compromise the capacity of the cell to endure oxidative stress.

Furthermore, this study revealed that Hdac5 and Hdac11 were significantly reduced in PS exposure in mice, while Kdm2a, Kdm3a, Kdm5a, Kdm6a, and Kdm6b were increased considerably in PS-NP mice compared to the healthy controls. These particular epigenetic modifiers are known to alter chromatin structure to control gene expression [64,65]. In particular, lipid metabolism is known to be regulated by Hdac5 and Hdac11 [66]. The disturbance of lipid metabolism carried out by PS-NPs might also be caused by the decrease in Hdac5 and Hdac11 [67], which are also reported to regulate inflammation. Therefore, the reduction in Hdac5 and Hdac11 can intensify the inflammatory response caused by PS-NPs [68]. Additionally, after being exposed to PS-NPs, the liver showed an increase in these particular KDMs, which may indicate epigenetic changes that affect liver function and general health [69]. The cellular stress reactions carried out by PS-NPs could be connected to the observed rise in KDMs [70]. Given that certain KDMs are linked to both liver fibrosis and cholesterol production, PS-NPs may have broad-ranging effects [71,72]. While the underlying causes are intricate and multifaceted, including inflammation, oxidative stress, disturbance of lipid metabolism, and epigenetic changes, further investigation is needed to comprehend these systems.

Finally, acetyl and non-acetyl histone acylation are frequently linked to active gene transcription [16]. The formation of histone acetyl/acylation states is hierarchical, as evidenced by the fact that several histone acetyl/acyl-transferases are frequently arranged into large complexes with histone acylation reader modules [73]. Among the methylations that have been studied more thoroughly are those in histone H3 Lys 4 (H3K4), H3K9, H3K27, H3K36, H3K79, and H4K20 [73]. H3K4, H3K36, and H3K79 have activating functions and are typically located in chromatin regions of transcriptionally active genes. Conversely, H3K9, H3K27, and H4K20 are primarily associated with the suppression of gene expression and frequently function as repressive markers [74]. Histone H3’s Lysine 18 residue undergoes post-translational modification to add a lactyl group, which is referred to as H3K18la or H3K18ac. P300/CBP acetylates histone 3 lysine 27 (H3K27ac) and histone 3 lysine 9 (H3K9ac), which are associated with active promoter and enhancer regions and facilitate transcription through epigenetic processes [75]. RNA splicing, elongation, and transcription development are among the signaling cascades coordinated by histone H3 trimethylated at lysine 4 (H3K4me1) [76], which is associated with substantial transcription elongation and enhancer activity, which leads to abnormally high gene expression, in contrast to other broad epigenetic features like super-enhancers [77]. Using ChIP-qPCR to quantify histone acetylated marks in the PS-NP-treated mice, it was discovered that, in comparison to the control mice, H3K9ac and H3K18ac were markedly downregulated at the target loci of *Hmox1* and *Sod3*. On the other hand, in the PS-NP-treated mice, histone methylation markers, including H3K4me3 and H3K27me3, were elevated at the target loci of *Hmox1* and *Sod3*. The notable rise in H3K9ac and H3K18ac indicates that the *Hmox1* and *Sod3* loci are more accessible, which permits higher transcription [78]. Occasionally, H3K4me3 is also detected at the loci of repressed genes, suggesting a more intricate regulatory function [79]. Polycomb Repressive Complex II (PRC2) deposits H3K27me3, which causes chromatin compaction and limits transcription factor access [80]. Consequently, it is likely that the downregulation of H3K9ac and H3K18ac and the upregulation of H3K4me3 and H3K27me3 in the PS-NP mice at the target loci of *Hmox1* and *Sod3* result in enhanced expression of these genes, improving the cells’ capacity to endure stress.

## 5. Conclusions

This study demonstrates that PS-NPs induce hepatic oxidative stress and injury in mice by suppressing the expression of key antioxidant genes, *Hmox1* and *Sod3*. These effects are mediated through the downregulation of NR RORγ activity and associated epigenetic alterations, including changes to histone modifications (H3K9ac, H3K18ac, H3K4me3, and H3K27me3) and transcriptional occupancy at the *Hmox1* and *Sod3* loci. Our findings underscore the importance of understanding the molecular mechanisms of nanoplastic toxicity and their potential health risks. Further research is needed to fully elucidate these pathways and validate molecular targets, which may inform future interventions to mitigate the pathophysiology induced by environmental plastic pollution in humans.

## Figures and Tables

**Table 1:** Product composition analysis: minimum values of nutrients in the basal diet (content per kg).

Items	Per kg
Moisture content, %	100 g
Crude protein	200 g
Crude fat	40 g
Coarse fiber	50 g
Coarse ash content	80 g
Calcium	10.18 g
Total phosphorus	12 g
Lysine	13.2 g
Methionine + cystine	7.8 g
Vitamin A	14,000 IU
Folic acid	6 mg
Vitamin A	120 IU
Vitamin D	1500 IU
Iron	120 mg
Manganese	75 mg
Zinc	30 mg
Selenium	0.1–0.2 mg

**Table 2:** Results of routine blood tests in mice following oral exposure to PS-NPs.

Items	Veh	PS-NPs
WBCs (K/µL)	9.345 ± 0.85 ^b^	15.51 ± 0.72 ^a^
Lymphocytes (%)	67.57 ± 3.74	70.85 ± 2.69
Monocytes (%)	5.57 ± 0.38 ^b^	9.32 ± 0.31 ^a^
Lymphocytes (10^6^/L)	3.54 ± 0.45	4.13 ± 0.56
Monocytes(10^6^/L)	0.88 ± 0.17 ^b^	2.48 ± 0.22 ^a^
RBCs (K/µL)	10.15 ± 0.22	10.02 ± 0.49
Hemoglobin (g/dL)	133.67 ± 7.92	135.17 ± 7.67
Hematocrit (%)	44.58 ± 3.28	39.88 ± 1.15
MCV (fL)	49.11 ± 1.30	39.97 ± 5.38
MCH (pg)	17.73 ± 0.43	17.47 ± 1.04
MCHC (g/L)	302.33 ± 6.05	307.83 ± 9.62
RDW-CV (g/dL)	15.25 ± 0.37	15.65 ± 0.52
RDW-SD (g/dL)	41.53 ± 1.77	38.85 ± 0.86
Platelets (10^9^/L)	439.50 ± 10.31 ^a^	357.00 ± 5.72 ^b^
MPV (fL)	9.21 ± 0.185	8.95 ± 0.26
PDW (%)	14.15 ± 0.85	14.01 ± 0.47
Plateletcrit (%)	550.00 ± 32.14	547.00 ± 23.16

Abbreviations: WBCs: white blood cells; RBCs: red blood cells; MCV: mean corpuscular volume; MCH: mean corpuscular hemoglobin; MCHC: mean corpuscular hemoglobin concentration; RDW-CV: red blood cell distribution width—coefficient of variation; RDW-SD: red cell distribution width—standard deviation; MPV: mean platelet volume; PDW: platelet distribution width. ^a,b^ Means with different superscripts in the same row differ significantly. The data are expressed as means ± SD (*n* = 6).

## Data Availability

RNA-seq data are available in the SRA under BioProject accession number PRJNA1305997.

## Supplementary Materials

The following supporting information can be downloaded at: https://www.mdpi.com/article/10.3390/biology15020135/s1. Figure S1: The GSEA depicting the enrichment of DEGs downregulated in the cell cycle, apoptosis, oxidative stress, lipid oxidation and inflammatory response pathways from PS-NPs versus vehicle in the mice liver; Figure S2: Western blotting analysis images.
