# Supplementary material for: Polystyrene Nanoplastic Exposure Causes Reprogramming of Anti-Oxidative Genes Hmox1 and Sod3 by Inhibiting Nuclear Receptor RORγ in the Mouse Liver
Source: Biology (Basel). 2026 Jan 13;15(2):135. doi: 10.3390/biology15020135 (PMC12837483; doi:10.3390/biology15020135)
Supplement: Supplementary file 1 [file biology-15-00135-s001.zip › biology-4036051-supplementary.pdf]

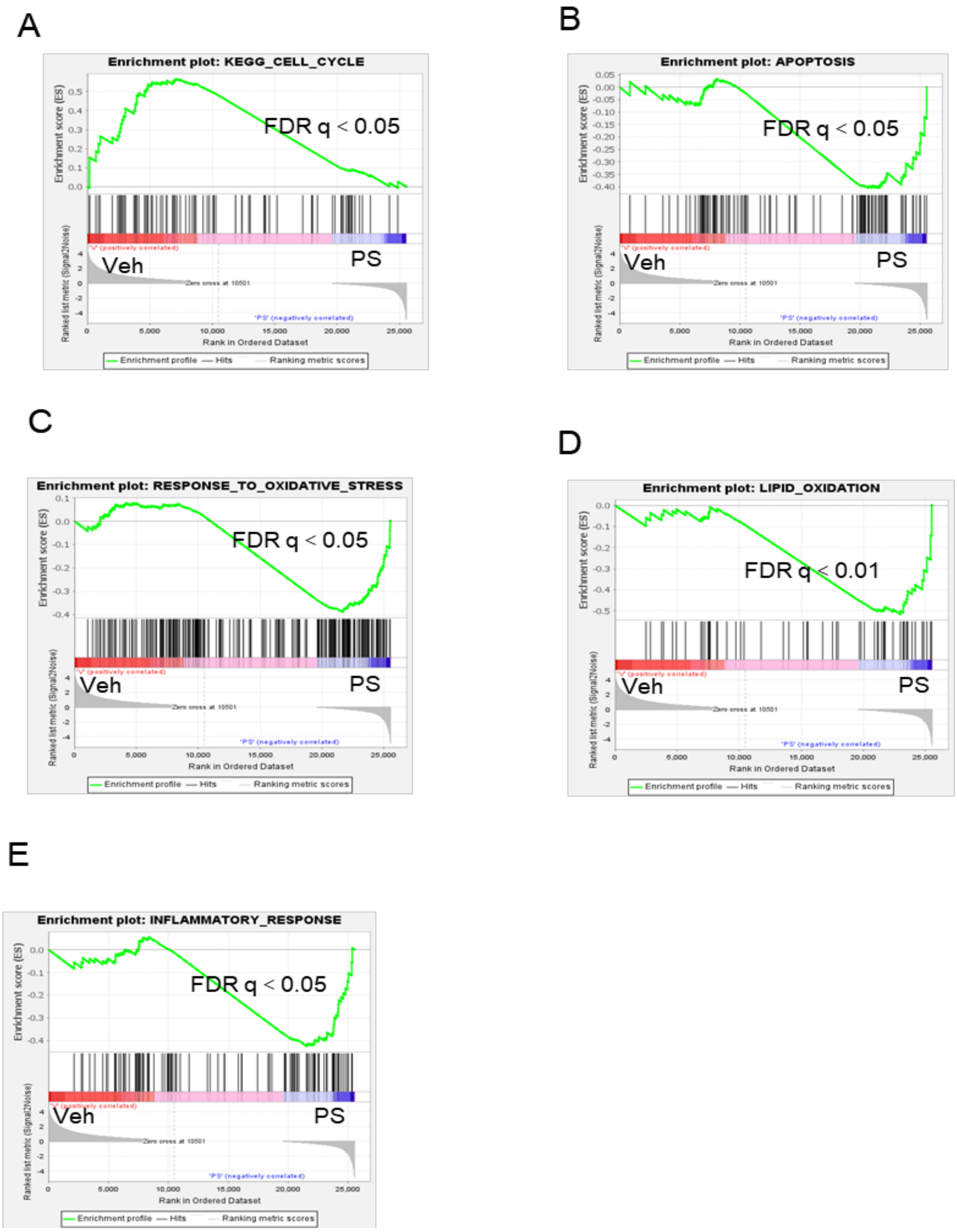

Figure S1 A-E The GSEA depicting the enrichment of DEGs downregulated in the cell cycle, apoptosis, oxidative stress, lipid oxidation and inflammatory response pathways from PS-NPs versus vehicle in the mice liver.

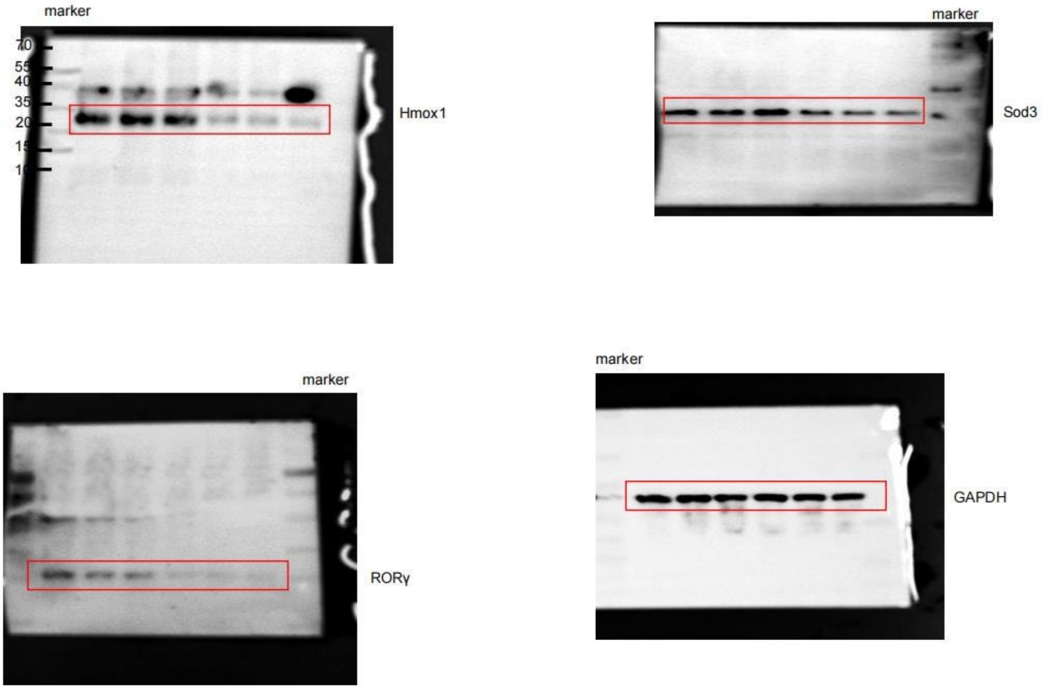

Figure S2 Western blotting analysis images
